# A narrative review of the physiology and health effects of burnout associated with veterinarian-pertinent occupational stressors

**DOI:** 10.3389/fvets.2023.1184525

**Published:** 2023-07-03

**Authors:** Michele A. Steffey, Dominique J. Griffon, Marije Risselada, Nicole J. Buote, Valery F. Scharf, Helia Zamprogno, Alexandra L. Winter

**Affiliations:** ^1^Department of Surgical and Radiological Sciences, School of Veterinary Medicine, University of California, Davis, Davis, CA, United States; ^2^Western University of Health Sciences, College of Veterinary Medicine, Pomona, CA, United States; ^3^Department of Veterinary Clinical Sciences, College of Veterinary Medicine, Purdue University, West-Lafayette, IN, United States; ^4^Department of Clinical Sciences, Cornell University College of Veterinary Medicine, Ithaca, NY, United States; ^5^Department of Clinical Sciences, North Carolina State University College of Veterinary Medicine, Raleigh, NC, United States; ^6^Evidensia Oslo Dyresykehus, Oslo, Norway; ^7^Merck & Co., Inc., Rahway, NJ, United States

**Keywords:** veterinary, burnout, occupational stress, wellbeing, practice management

## Abstract

Chronic workplace stress and burnout are serious problems in veterinary medicine. Although not classified as a medical condition, burnout can affect sleep patterns and contributes to chronic low grade systemic inflammation, autonomic imbalance, hormonal imbalances and immunodeficiencies, thereby increasing the risks of physical and psychological ill health in affected individuals. Cultural misconceptions in the profession often lead to perceptions of burnout as a personal failure, ideas that healthcare professionals are somehow at lower risk for suffering, and beliefs that affected individuals can or should somehow heal themselves. However, these concepts are antiquated, harmful and incorrect, preventing the design of appropriate solutions for this serious and growing challenge to the veterinary profession. Veterinarians must first correctly identify the nature of the problem and understand its causes and impacts before rational solutions can be implemented. In this first part of two companion reviews, burnout will be defined, pathophysiology discussed, and healthcare and veterinary-relevant occupational stressors that lead to burnout identified.

## 1. Introduction

Stress was considered the most critical issue facing the veterinary profession by 92% of 2,874 respondents in the 2020 Merck Animal Health Veterinarian Wellbeing Study (1). In a survey of 889 North American veterinarians, 43% answered “Often” and 9% answered “Always, ” in response to the question “how often have you felt distressed or anxious about your work?” demonstrating the high level of distress in the daily lives of many members of the profession (2). According to the AVMA, 40% of veterinarians are considering leaving the profession and the expressed top reasons for wanting to leave are a lack of work-life balance followed by challenges of stress and mental illness (3). Impacts of chronic stress and burnout have been documented across a wide range of professions, although healthcare professions such as medicine and veterinary medicine exhibit high rates relative to the general population and other occupations. The overall burnout rate of veterinarians reported in the past 5 years has ranged from 27 to 89% (1, 4–8) with notable variation amongst different veterinarian cohorts. The severity of reported veterinarian burnout scores was nearly 40% higher than those of a comparable group of physicians (1). This is notable, as longitudinal studies have similarly and consistently found greater occupational distress among US physicians compared with the general US workforce, even after adjustment for age, gender, work hours, and level of education (in 2015, OR = 1.97, 95% CI = 1.80–2.16, *p* < 0.001) (9, 10). The 2022 physician burnout and depression report documented an overall US physician burnout rate of 47% nationally, with a range from 33 to 60% reported across different medical specialties (11). Seventy-six percent of physicians report that the impact of burnout on their daily lives is moderate (22%) or severe (54%), and that most are either more (50% of men, 60% of women) or equally (42% of men, 31% of women) affected currently compared to the quarantine months of COVID-19 in 2020 (11). Sixty-eight percent of physicians indicate that burnout has adversely affected their personal relationships (11). The depth and breadth of data on burnout are better stratified for physicians compared to veterinarians, however the demographics are similar: ~50% of veterinarians report moderate to high levels of burnout (6, 12), women veterinarians exhibit higher burnout scores compared to men (*p* < 0.001) (6, 13, 14), less experienced veterinarians exhibit higher burnout rates compared to those with more experience (6), and greater burnout is seen among those carrying high educational debt (6). The professional education, structure, culture, and daily experiences of veterinarians and physicians share many similarities, including parallel causes and trajectories of burnout. In general, the healthcare professions including physicians, nurses, and others as a group are demonstrated to be particularly prone to burnout, and many risk factors appear to be shared within these professions (15–21). This may also imply that risk reduction and prevention strategies can be applied across the spectrum, and veterinary medicine should look carefully at the relevant burnout risks, impacts, and solutions that have been identified in human healthcare.

Individuals enter veterinary school and practice fully anticipating a challenging and demanding profession. Stress is expected owing to the intense nature of the work, but is often unnecessarily amplified by poorly designed, entrenched professional structures and systems. In 2021, only 41% of all veterinarians and 24% of veterinarians under 34 years of age would recommend the profession to a friend or family member (compared with 75% of veterinarians in 2005) (22). Sixty five percent of veterinarians feel that the workload is consistently excessive, and far too many have experienced depressive episodes (31%) or suicidal ideation (17%) since their graduation (1, 22–24). This chronic stress and burnout contribute to escalating rates of distress and attrition from the profession. Increasing burnout is a threat to the delivery of veterinary care, and this creates a self-perpetuating, escalating cycle. The main reasons cited for early exit of the profession are mental health (33%) and work-life balance (27%) (25). Veterinarians are approximately twice as likely as other healthcare professionals and up to 4 times as likely as the general U.S. population to die by suicide (26–30). Approximately one in 15 US physicians reported suicidal ideation within a 12-month period, a higher prevalence than a general population of US workers (7.1 vs. 4.3%, *p* < 0.001) (31). Data on deaths in the U.S. from 1979 through 2015 indicates that 79% (313/398) of veterinarians who died by suicide during this period were in clinical practice occupations, and of the veterinarians in clinical positions who died by suicide 75% (226/300) worked with companion animals (29). A strong relationship of burnout to suicidal ideation (independent of depression) has been established in studies of medical students (32), physicians (33), and veterinarians (12). Burnout appears to be an important mediator in understanding veterinarians' suicidal tendencies, and organizational interventions that are effective in reducing burnout may also reduce the risk of suicide (34). Testifying to the growing awareness of these problems in the veterinary profession, the recent literature on veterinarian mental health, occupational stress, and burnout is growing (1, 4, 6, 35).

Occupational stress is a complex problem. Solutions for workplace stress, poor mental health, and burnout in veterinary medicine are needed, but the peer-reviewed veterinary literature on these topics too commonly focuses on reporting individual traits as predictors of coping difficulties and advising personal wellness techniques as methods of resolution (4, 14, 36–40). The identification of individual traits (such as personality type) as causative factors is of limited impact and does not address fundamental workplace problems. The 2022 physician burnout and depression report detailed that only 34% of 13,000 physicians representing 29 specialties responded yes to the question: “Do you feel your personality type is a contributing factor to burnout?” (11) Wellness approaches do not provide correction of the fundamental issues of veterinary practice that lead to burnout. While the positive impacts of exercise, nutritional self-care and wellness practices of mindfulness, gratitude, and resiliency training on stress management are inarguable, they act as short-term coping strategies. Decades of research suggest that interventions targeting only individuals are far less likely to have a sustainable impact on employee health than systemic solutions (41, 42). The issue of veterinary burnout will not be adequately addressed if the profession continues to rely predominantly on the self-care practices of individual veterinarians (43, 44). More foundational change in veterinary education and practice systems will be required to ensure the sustainability of the profession under current and future societal conditions.

The objectives of this narrative review are to discuss core causes, and to explore sources of burnout relevant to the veterinary profession. In a companion article (45) we will describe the demographics of burnout in the veterinary profession and discuss the impact of burnout on the function of the veterinary workplace before extrapolating potential strategies. These reviews are intended to supplement existing reviews on veterinary wellbeing (30, 39, 46–52) as a foundation for well-informed discussions within our profession. It should be noted that burnout occurs in a wide variety of workplace environments (53–55), and that all members of the veterinary education and practice teams may be vulnerable to occupational stress and burnout (13, 22, 35, 56–62); their experiences are not minimized. However, this review and its companion will focus on the subset of issues and conditions most relevant to clinically practicing veterinary clinicians, house officers (post-graduate interns, residents, and clinical fellows), and students.

### 1.1. Search strategy

One author (MS) searched PubMed and Google Scholar for “veterinary and burnout” and “occupational stress and veterinary,” “wellness and veterinary” and “mental health and veterinary”. Several combinations including the terms “burnout”, “occupational stress”, “healthcare”, “physician” “surgical” and “medical” were applied to identify relevant publications between 2017 and 2022. Manual scoping of results focused on original research manuscripts, meta-analyses, and systematic reviews. Original research obtained by a manual scoping of references in articles that were identified in the initial search was then incorporated, as well as original research identified by date-unconstrained focused topic searches as needed to improve conceptual understanding. The eligibility criteria included: all relevant literature on veterinary burnout, relevant supporting literature on the pathophysiology of burnout, literature on quantitative impact of burnout on occupational performance within clinical human healthcare, peer-reviewed articles written in English, and full-text articles. Studies of non-occupational causes of burnout were excluded.

## 2. The culture of veterinary medicine

Culture refers to the shared and fundamental beliefs, normative values, and related social practices of a group that become so widely accepted that they are implicit and no longer scrutinized (63, 64). Manifestations of culture within the workplace including overt policies and incentive systems are often mistaken for culture but should better be conceived of as organizational climate (which can be altered through leadership choices) (63). Culture is more expansive and deeply rooted than climate, and manifests as symbols (the behaviors, heroes and rituals of a culture; e.g., the late Dr. James Herriott is a hero to many, and a common symbol of the profession), espoused values (e.g., the Veterinarian Oath recited at a graduation ceremony), and tacit assumptions (the unwritten rules that drive one's daily behavior; e.g., the belief that veterinarians should always prioritize the best interest of the patient over financial or logistical concerns) (63). While there is a preponderance of good in the culture of veterinary medicine, seriously problematic sub-components can and do still coexist, and can be noted in the divergence between the profession's stated values and artifactual behaviors (63). For example, while the value that “self-care is important,” may be proclaimed, in reality, the artifactual behavior is that too many veterinarians either choose or are required by workplace policies or tacit assumptions to work under unrealistic conditions or expectations, and individual health needs are ignored. This reveals that culturally there are expectations for veterinarians to be superheroes, impervious to stress and fatigue, or beliefs that personal health is subservient to work. This cognitive dissonance manifests in practice organizational structures, policies, and unspoken expectations. When a part of a culture, it is easy to become blind to these inconsistencies. Changing the culture of veterinary medicine and the climate of the workplace will require holding dearly held cultural beliefs up to scrutiny, and honestly acknowledging the unhealthy norms, blind spots, and tacit assumptions that contribute to the problems at hand.

## 3. What is burnout?

“Burnout” is often used in the common vernacular to describe an unspecified tiredness and/or stress; however, the term has specific and defined application related to long-term effects of work-related stress (Table 1). Historically, burnout has been described as a work-related syndrome of physical and emotional exhaustion secondary to chronic occupational stress, and characterized as mild, moderate, or severe (65). Burnout has also been described based on clinical subtypes. The frenetic subtype is typical of work overload and workers who are involved, highly dedicated, and tend to work intensely until exhaustion (66, 67). It has been related to a coping style based on attempts to solve problems actively, and for which individuals use a high number of working hours/week or are involved in different jobs at the same time (66, 67). The underchallenged subtype is typical of monotonous and unstimulating professions, with repetitive, mechanical, or routine tasks that do not provide worker satisfaction or personal development (66, 67). It is associated with escapist coping styles, high levels of cynicism, and workers who become indifferent and bored (66, 67). The worn-out subtype is characterized by feelings of hopelessness, lack of control over work results, and lack of recognition of their invested efforts, such that affected individuals respond to difficulty with neglect and abandonment (66, 67). A progressive deterioration from the frenetic to the under-challenged or worn-out subtype has also been suggested (68).

**Table 1:** Definitions of relevant wellbeing, mental health, and burnout concepts.

**Definitions**
**Anxiety disorder** – An emotional state characterized by disproportionate feelings of tension, recurring, intrusive thoughts or concerns and physical changes like increased blood pressure or heart rate
**Burnout** – A workplace syndrome of physical and emotional exhaustion secondary to chronic occupational stress, with a spectrum of severity and symptomology. Distinct from depression and anxiety disorders, but occurrence and symptoms may overlap. Differs from compassion fatigue in that burnout usually stems from excessive responsibilities or time at work
**Caregiver burden** – The chronic stress or exhaustion due to the physical, emotional and mental strain of caregiving
**Clinical burnout** - Burnout severe enough to cause secondary physical and mental health conditions
**Compassion fatigue** – A form of secondary stress resulting from the physical, emotional, and psychological impact of helping others and exposure to the pain and distress of others. Often mistaken for burnout but related specifically to the diminishment of the empathetic response over time. It has been recently suggested by Perret et al. (14) that a more accurate descriptor of this condition would be “empathic distress”
**Cynicism/Depersonalization** - A dysfunctional, self-protective, coping mechanism for chronic work stress and exhaustion characterized by dehumanized and/or cynical attitudes toward the recipients of one's services. May include clients, colleagues, or institutional structures
**Depression** - A medical illness characterized by feelings of severe despondency, sadness, and/or inadequacy lasting more than 2 weeks, often accompanied by lack of energy and disturbance of appetite and sleep, and a loss of interest in activities once enjoyed
**Emotional exhaustion** – The decreased emotional energy as a result of excessive personal or work demands
**Hidden curriculum** - The implicit, unwritten, and sometimes unintended social and cultural norms, behaviors, values, rules, and expectations unofficially conveyed in an educational setting
**Job demands** - All physical, psychological, social or organizational aspects of a job that require physical, cognitive and/or emotional effort
**Job strain** – Psychosocial workplace stress that is comprised of high job demand and low job control
**Moral injury or distress** – The emotional state that arises when one identifies an ethically correct action to take but feels powerless or constrained from taking that action. This is distinct from burnout but not mutually exclusive and these conditions can occur concurrently. Moral injury may contribute to the development and progression of burnout
**Pathological altruism** - Behaviors that attempt to promote the welfare of others but themselves have damaging long-term consequences for the caregiver
**Presenteeism** - The behavior of coming into work when unwell/ill (applies to both physical and significant mental illness)
**Professional efficacy** - Job satisfaction and feeling of competence and successful achievement in one's work
**Role ambiguity** - Whereby individuals are uncertain about the scope of their duties, authority, allocation of time, and relationships with others
**Role conflict** - Emotional conflict arising when contradictory, competing or incompatible demands are placed on an individual in the fulfillment of their job or position
**Resilience** - The ability to “bounce back” from negative emotional experiences and to adopt flexible solutions to the changing demands of stressful experiences
**Somatization** - The physical manifestation of psychological symptoms that are insufficiently treated. These physical symptoms are not consciously controlled and may be life-disrupting or cause significant distress. Somatization examples include stress-related migraines or GI disorders
**Secondary traumatic stress** - a negative feeling driven by fear and trauma experienced indirectly through hearing details or witnessing the aftermath of a trauma experienced by another person. In veterinary medicine, this is commonly a work-related trauma and can be related to caring for and euthanizing suffering animals or providing emotional support and comfort to clients. Symptoms include difficulty concentrating, irritability, sleep disturbances, intrusive thoughts, and traumatic memories, and can be similar to post-traumatic stress disorder. Unlike burnout, these symptoms are usually rapid in onset (6)
**Work compression** - The expectation that a fixed amount of work is completed, but within fewer hours
**Workplace bullying/mobbing** – Repeated, less favorable treatment of one person by another in the workplace (a supervisor, subordinate, co-worker, or colleague) by actions that may be overt or covert and includes behaviors such as excluding or isolating individuals from opportunities/information/interaction with others, using undermining or demeaning language, creating undue pressure and stress, social humiliation, aggression, and harassment. Bullying is generally performed by one individual; mobbing describes multiple individuals targeting the same person
**Workplace post-traumatic stress disorder (PTSD)** – Long lasting emotional, cognitive, and physical challenges related to negative, abusive, or traumatic workplace stressors or toxic work environments, with symptoms that include hyperarousal to stressors, flashbacks, nightmares, severe anxiety, and uncontrollable thoughts about the stressor

As of May 2019, burnout was incorporated in the 11th revision of the International Classification of Diseases (IDC-11), although it is still classified as an occupational phenomenon, not a medical condition (69). The experience of burnout can also include physical signs such as fatigue and somatization (Table 1), with eventual development of secondary chronic medical conditions (70). While burnout is characterized as an occupational syndrome, it can be causally and bidirectionally related with true mental and physical illness. It is a vulnerability that can promote the development of depression and/or anxiety and may be exacerbated or accelerated in the presence of pre-existing poor mental health (71). Burnout severe enough to cause secondary physical and mental health conditions is an indication for health professional assistance and has been referred to as “clinical burnout,” “work-related mental health impairment,” or “stress-related exhaustion disorder” (42, 72). Vicarious traumatization, secondary trauma, moral distress, compassion fatigue/empathic distress, anxiety disorder, and depression (Table 1) are other related, but distinct, stress-based experiences that can contribute to emotional exhaustion or depersonalization but should be distinguished from burnout (56, 62). Current research describes the relationship between compassion fatigue/empathic distress and burnout as overlapping, but it is important to recognize that these are distinct concepts. Burnout describes a general reaction to work-related environmental stress that can be experienced by employees in any occupational field, and compassion fatigue, empathic distress, moral distress, or secondary traumatic stress directly relate an employee's relationship with the specifics of the work that is being undertaken (13).

The definition of burnout varies across studies, but most researchers follow Maslach's three-dimensional concept that burnout consists of three domains of emotional exhaustion, cynicism/depersonalization, and a low sense of professional efficacy or accomplishment (Table 1) (73). A number of screening tools for identification of burnout are now available, adapted for the specifics of the language and culture of the population studied, although the Maslach Burnout Inventory (MBI) is generally considered the standard assessment for burnout and most frequently used for this purpose (74). Although exhaustion usually precedes cynicism, subsequently affecting professional efficacy, burnout can be present with an extreme score in just one of any of these 3 domains (60). More recently, it has been suggested that characterizing individuals on a spectrum of engagement to burnout using the MBI may be more accurate, and four clusters along this spectrum were identified in veterinarians: engaged (10.8%), ineffective (18.9%), overextended (29.6%) and burnout (40.7%) (8). Results of this study indicated that most participants (89%) had one, or a combination, of high exhaustion, high depersonalization, and low professional efficacy (8). Other psychometric tools reported in the veterinary literature to characterize occupational stress, wellbeing, and mental health have included the Professional Quality Of Life Survey (ProQOL; a measure of compassion fatigue that incorporates two subcomponents of burnout and secondary traumatic stress), the Mayo Clinic Physician Burnout and Wellbeing Scale, the Generalized Anxiety Disorder Scale, the Patient Health Questionnaire, The Optum Short Form-8 Health Survey, Kessler Psychological Distress Scale, the Connor-Davidson Resilience Scale, and the Hospital Anxiety And Depression Scale, although many other survey instruments exist (1, 4, 6, 7, 40, 75).

Neuroscience provides evidence for burnout as a distinct entity. Compared to control subjects, individuals suffering from burnout exhibit diminished neurophysiological responses to stimuli, with the cognitive deficit situated at the level of task performance (76, 77). Task performance is a complex interplay between sensory inputs, long term memory, and working memory. Human sensory and long-term memory systems are both capable of dealing with large volumes of information, however in comparison, the capacity of working memory is much more limited (78). Working memory is further directed by the attention or metacognitive capacity of each individual (78). Cognitive load represents the overall burden on working memory experienced by an individual, as a sum of the various sensory or task-specific demands, as well as psychological and emotional factors (78). Total task cognitive load may be further subdivided into three components: intrinsic load (the inherent task complexity), extrinsic load (the load imposed by the way in which the information is presented), and germane load (the workload of learning or creating mental models or associations) (79). An individual's capacity for cognitive load is fixed and not amenable to change, although specific training may help improve metacognition (78). When task completion requires coordination of so many independent elements that working memory is exceeded, cognitive overload occurs (78). Interruptions contribute to task overload and result in lower performance on complex tasks, an effect exacerbated by the interruptions frequency and the dissimilarity of content between the primary and interruption tasks (80). When an individual approaches the point of cognitive overload, they may begin to demonstrate deterioration in quality or quantity of work compared to their own previous standard, as well as exhibiting behaviors similar to depressive symptoms (78). It is postulated that the phenomenon of burnout may be a self-protective neuropsychological response to attempts to function beyond a fixed capacity (78). Results of a recent study of physician intensivists suggest that alarming numbers of clinicians may be living at or near the brink of workload and capacity imbalance (81).

## 4. Why does burnout occur?

**Figure 1:**
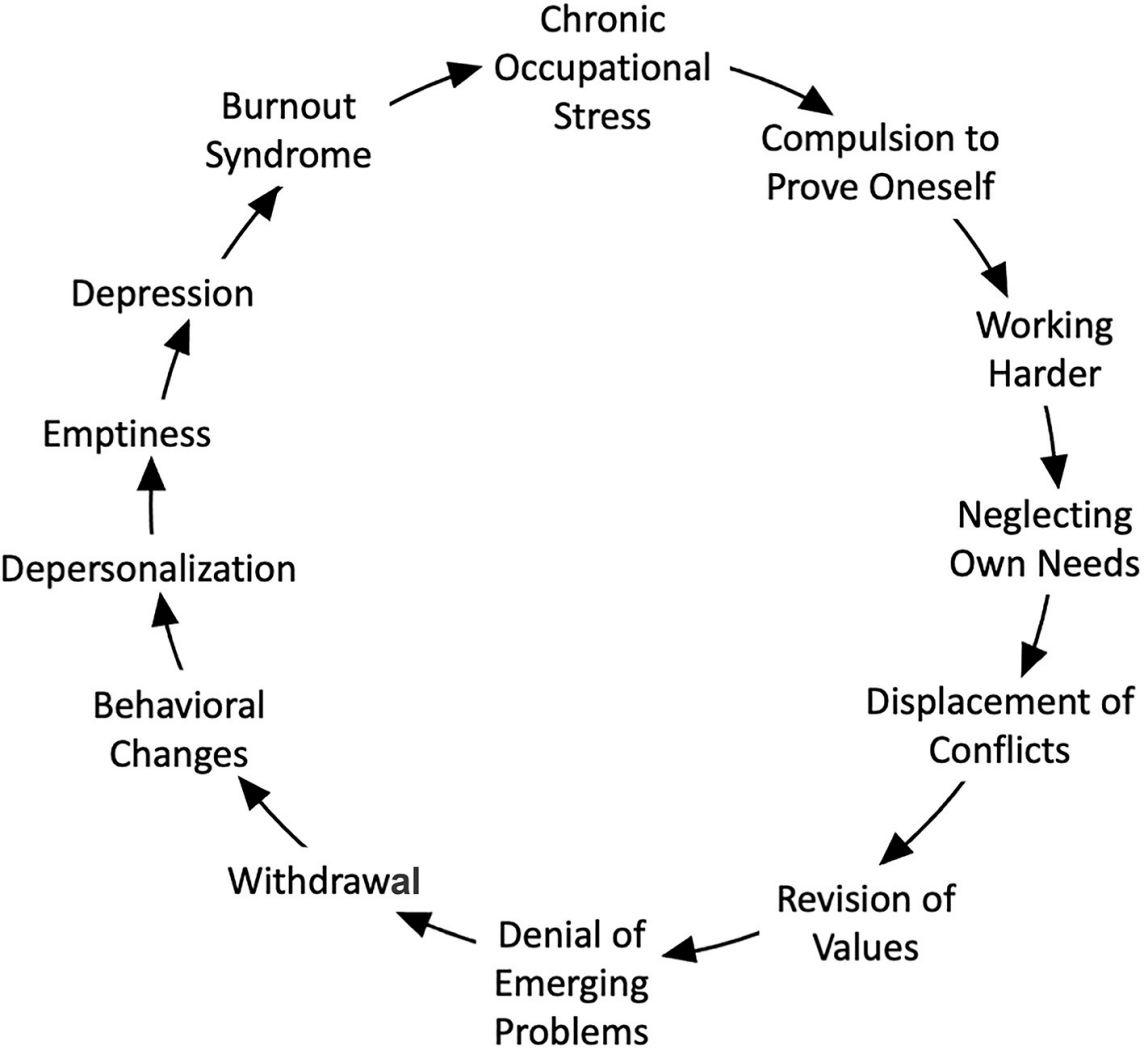
A multi-step model for the development of burnout adapted from concepts described by Freudenberger and de Hert (84, 85).

Burnout is the result of prolonged, unresolvable stress at work and is fundamentally caused by a chronic mismatch between the demands of the job and the resources of the worker (73, 82). This work-focused, reversible syndrome results from an enduring adaptive failure and is not the same as non-morbid, acute job stress, nor is it alone considered a mental disorder or disability (83). The etiopathogenesis is multifactorial and complex, as evidenced by the variety of causative factors and psychological explanatory models of burnout (84). De Hert further summarized Freudenberger's work on the development and progression of burnout at an individual level as a 12-step gradual process (Figure 1) (84, 85).

In contrast with burnout, individuals affected by short-term work-related stress describe a clear relation between a stressor and symptoms experienced within 3 months (86). Individuals that develop burnout often ignore stress symptoms for several years and commonly describe that living a stressful life was a normal condition for them (86). Some may not even be actively aware of the level of stress in their lives, until they reach a point of collapse, often after exposure to a minor stressor or metaphorical straw that breaks the camel's back (86). Individuals who develop burnout are rarely inclined to seek help. Instead, they tend to persevere, with ongoing efforts to perform or achieve at high levels despite the overwhelming difficulties, without complaining or asking for social support. Perseverance is a desirable trait when one has control over one's situation, but it is not an adaptive response when control of one's situation is lacking (86). Humans can endure considerable amounts of stress as long as stressful periods alternate with periods of recovery. Burnout starts with insufficient opportunity to recover from physiological stress reactions (86). It can be further sub-categorized on a spectrum from early/mild (when signs are not incapacitating) to severe or medically significant (when exhaustion and impaired performance in work and personal life justifies professional help) (72). Burnout has been established in human healthcare as the predictable and inevitable consequence of (1) the way that medical education and practice are organized, and (2) the maladaptive behaviors initially engraved as coping mechanisms in early career and subsequently reinforced by workplace culture and practice management choices (43). These risks should be equivalently recognized by the veterinary profession.

## 5. Pathophysiology and effects of burnout in individuals

Burnout is a known risk factor for poorer physical health, increasing the risk for early onset of age-related diseases and premature death (87). In a study of 7,396 people < 45 years of age at onset, conducted over nearly 11 years, individuals demonstrated a 35% increased risk in mortality (95% CI = 1.07–1.71) with each one-unit increase in total burnout score (87). The mechanisms driving these effects are not fully elucidated but are linked to uncontrolled chronic stress, and center on the sequelae of repeated and prolonged activation of the sympathoadrenal and hypothalamic-pituitary-adrenal stress systems (88–91). The physiologic effects of burnout resemble those of uncontrollable stress and fatigue, and result in impaired regulation of thought, action, and emotion; effects not seen with controllable stress (92). In functional MRI studies, patients with clinical burnout tend to under-recruit prefrontal cortical areas when performing tasks involving memory and executive functions; this frontal hypoactivation occurs in the presence of apparently intact functional networks (93). Emotional instability results from a functional disconnection between the amygdala and the medial prefrontal cortex, as documented with positron emission tomography: affected individuals lose the ability to inhibit stress responses of the limbic system from the higher cortical centers (94). With chronic exposure to stress, connections within the prefrontal gray matter are lost, but can be restored when stress is relieved (92).

The stress fight-or-flight response has been proposed to be the default physiologic baseline that operates continuously unless actively suppressed by a sense of safety or calm (90). This reactive, large-magnitude response of physiological systems to short-term threat is an evolutionary adaptation to immediate danger. However, prolonged responses (caused by anticipatory activation or delay/failure to recover) lead to imbalance, overuse, and dysregulation of the chronic autonomic nervous system (90). This default response must be actively dampened to prevent deleterious effects, and growing evidence supports the concept that this necessary inhibition is accomplished by individual recognition of safety (90). It is a failure to receive safety signals that dampen the fight-or-flight response combined with insufficient opportunities for rest and recovery that appears to be fundamental to the development of burnout, rather than active perceptions of threat that positively stimulate fight-or-flight responses (90). This mechanism explains the causative roles of workload, toxic work environments, circadian disruptions, and other chronic work-related stressors with regards to burnout.

### 5.1. Insufficient rest

The American Association of Sleep Medicine has identified clinician burnout as an underappreciated public safety issue, yet sleep loss is often overlooked as a contributing factor to this problem (95). Chronically disrupted, impaired or insufficient sleep, insufficient recovery time, and circadian disruptions are all consistently highlighted as major sources of chronic work stress and dissatisfaction, anxiety, and general health problems, and are strongly associated with burnout both as a cause, and as an effect (48, 49, 72, 96–99). Changed sleep physiology is one of the symptoms of burnout but is also a mechanism in its pathophysiology, contributing to the cardinal symptom of fatigue. Patients with high burnout scores experience fatigue resulting from trouble falling asleep, early awakening, sleep fragmentation, decreased sleep efficiency (time in bed spent actually asleep), increased sleep latency, and/or non-refreshing sleep (98). These signs are accompanied by decreased slow wave activity (98). The deleterious effects of burnout on sleep seem independent of co-morbid major depression, supporting that sleep problems are independent concomitant symptoms in burnout (98). Sleep insufficiency is associated with job strain and burnout in a dose-dependent manner; development or worsening of insomnia and sleeping difficulties may precede and predict burnout (98, 100).

In a recent study of disordered sleep and burnout, 29% of 1,074 faculty and staff physicians employed in a teaching hospital system screened positive for at least one sleep disorder (92% of which were previously undiagnosed) (101). A positive screening result for a sleep disorder was associated with a nearly 4-fold increased odds of occupational burnout in this cohort (OR = 3.67; 95% CI = 2.75–4.89) (101). Insufficient sleep affects the metabolic and physiological activities of the prefrontal cortex, correlating with observed cognitive deficits (102). Reduced effectiveness of top-down attentional control occurs under conditions of sleep restriction, and when the task environment requires flexibility, performance suffers dramatically, which helps to explain performance deficits that are not readily explained by lapses in vigilant attention (103). Indeed, sleep loss and fragmentation are themselves associated with impaired executive function and psychomotor performance (98). Sleep-deprived individuals show emotional disinhibition, heightened anxiety, and impaired emotional responsivity, feel lonelier, and behave in a less prosocial manner (104, 105). This predisposition to loneliness justifies special consideration as this feeling is self-reinforcing and has been identified as a greater risk factor for premature death than obesity (106). If an individual is perceived as lonely, others will frequently disengage from interacting with that person, perpetuating the cycle of social isolation that worsens burnout and its associated health impacts (106). Notably, improving the magnitude and quality of sleep plays an important role in burnout convalescence and allowing return to work (98, 100).

Studies in aviation have demonstrated that the number of consecutive workdays and the presence and number of consecutive late finishes by themselves impact fatigue and stress levels (107). Longer workdays or work weeks are associated with a cumulative insufficient recovery time from work, and result in poorer mental health status, increasing levels of anxiety and depression symptoms, and sleep disturbances (108). Physicians who work over 55 h/week are more than twice as likely to report development of mood disorders (OR = 2.05, 95% CI = 1.62–2.59, *p* < 0.001) and suicidal ideation (OR = 2.00, 95% CI = 1.42–2.81, *p* < 0.001) compared to those working 40–44 h/week (109, 110). In a recent meta-analysis, 13/17 studies evaluated found that hours worked/week was a predictor of burnout, psychiatric morbidity, and diminished professional efficacy, career satisfaction, and quality of life (111). In a study of 1,560 physicians, working long hours (40–60 h/week) was more likely to be associated with burnout compared with working ≤40 h/week (OR = 1.58, 95% CI = 1.15–2.17), with an even stronger association with burnout exhibited when working >60 h/week (OR = 2.59, 95% CI = 1.28–4.08) (112). Among those physicians who worked over 60 h per week, 53% of respondents were in the upper tertile of burnout (112). In another study, a non-linear dose-response relationship between work hours and burnout was identified: compared with working 40 h/week, the odds of work-related burnout doubled when the work week exceeded 60 h, tripled at >74 h/week, and quadrupled at 84 h/week (113). In addition to mental health effects, this type of overwork has impacts for physical health. In a recent meta-analysis that included data from 768, 751 workers, an 17% increased risk of ischemic heart disease and 35% increased risk of stroke was identified in people working ≥55 h/week compared to a typical 35–40-h week (114).

### 5.2. Physical impacts

Signs of fatigue generally present early in individuals affected by burnout but are typically not taken seriously (98). The chronic stress that results in burnout exacerbates the associated low grade systemic inflammation and autonomic imbalance caused by chronically elevated sympathetic tone. Autonomic imbalance increases the risks of hypertension, coronary heart disease, and type 2 diabetes mellitus, among other diseases (90, 91). In fact, a meta-analysis of the literature demonstrated that the relative risk of cardiovascular events associated with burnout is at least equal to that conferred by more established and well-known factors such as age, body mass index, smoking, blood pressure, and lipid levels (115). Job strain is associated with higher systolic and diastolic blood pressures with a non-dipping pattern conserved throughout working hours, but also at home and even while asleep (when blood pressure should normally be reduced) (90). This chronic lack of blood pressure reduction at night can lead to end organ damage and mortality (90). Stress also causes chronic dehydration in humans, affecting fluid shifts between the intracellular and extracellular compartments and the renin-angiotensin-aldosterone system, thereby promoting the development of secondary renal disease (116).

Chronically stressful conditions cause hypertrophy and hyperplasia of the adipocytes, alter the secretion of adipokines, and cause attraction and activation of stromal fat immune cells; these dysfunctions lead to carbohydrate intolerance, insulin resistance, type 2 diabetes mellitus, dyslipidemia, non-alcoholic fatty liver, and hypertension (116). Lower prolactin, thyroid stimulating hormone and total triodothyronine (T3), as well as higher testosterone levels have been documented in women with elevated burnout scores compared to healthy controls (117). Chronic stress has been implicated as both a cause of and a factor that exacerbates existing polycystic ovarian syndrome, the most common disorder in women of reproductive age, and one that seriously impacts both general and reproductive health (118). Psychological stress is harmful to sperm and semen quality, contributing to infertility in men (119). Chronic stress directly affects the gastrointestinal tract by facilitating dysbiosis or microbial imbalance, increasing gut barrier permeability, and promoting systemic inflammation and immune dysregulation, and conversely the composition of the gastrointestinal flora itself plays a role in the pathology of stress-related disease (120, 121).

Long-term work-related psychosocial stress, characterized by low job control and high job strain, is associated with increased risk of dementia and Alzheimer's disease later in life in multiple studies (122, 123). Stress-related inflammation markers have been identified as predictive factors for loss of bone mineral density, directly due to hypercortisolemia and indirectly via the vicious cycle of constant subclinical inflammation, increased pro-inflammatory cytokines, and disruption of bone remodeling balance (116). The combination of chronic stress, hypercortisolism and systematic low-grade inflammation affects muscle mass, an important predictor of human health (116). High psychological and social stress is also associated with decreases in naïve and increases in terminally differentiated T cells, impacting immune health, reducing infection resistance, and potentially contributing to generalized elevated risk for poor health (90, 124). High levels of burnout are robustly associated with increased risks for infectious disease, including COVID-19 (125).

### 5.3. Cognitive and mental health impacts

Individuals affected by clinical burnout commonly complain about cognitive deficits, affecting problem-solving, learning, attention, focus, and failing to keep important information such as names or appointments (126, 127). This subjective mental fatigue may persist up to 3 years after diagnosis even with remediation attempts, demonstrating long-lasting consequences on the daily functions of affected individuals (128). Importantly, a national study of over 16,000 internal medicine resident physicians found a strong and negative correlation between burnout and medical knowledge as measured by standardized tests (129). The effect size of the association between burnout and medical knowledge was roughly equivalent to an entire year of residency, and lower medical knowledge scores persisted on longitudinal analysis (129). Complex higher cognitive functions such as attention, concentration and working memory, focus and decisiveness are impaired in individuals with burnout (130). Neuropsychological testing of burnout-affected individuals further supports the presence of performance deficits in cognitive tasks requiring attention and visuo-spatial constructional ability (131), as well as executive control tasks, attention span, working memory, learning and episodic memory (132). However, cognitive impacts are not identified in individuals with non-clinical burnout and exhibit a great deal of individual variability in those with clinical burnout (133, 134). In another study, higher burnout scores correlated with increased brain activation when performing tasks requiring attention, indicating that individuals with burnout must mobilize more brain resources to complete these cognitive tasks (135). Experimentally, the cognitive performance of affected patients could not be increased through a motivational intervention; inferior performance on tests of attention and reaction time could not be improved by false positive feedback or by monetary incentives (136, 137). Overwhelming evidence supports the neurobiological basis of these signs, independent of personal choices.

Individuals affected by chronic stress tend to reduce situational complexity by applying more rigid ways of problem solving and cognitive simplification (86). This “shortcut” in processing information may lead to incorrect interpretations that an employee is exhibiting maladaptive personality traits rather than symptoms of burnout (86). A cycle of attributed blame, and efforts to fix the employee exacerbate stress in the affected individual, rather than addressing the suboptimal and stressful environment that fundamentally drives the physiologic changes and observable symptoms (86). For effective personnel management in professions with high rates of burnout, expression of a rigid, maladaptive interpersonal style should be investigated as either a potential cause or a result of chronic stress, as these fundamental issues are best addressed in very different ways (86).

Behavioral manifestations of burnout unfold over time, and mental health impacts typically result from a combination of chronic stress and job dissatisfaction (138). Chronic personal distress erodes professionalism (139, 140). Earlier manifestations of burnout may include emotional instability characterized by uncontrollable mood swings and expression of intense and overwhelming emotions. Over time, psychosocial manifestations can transform into true mental health disorders, observable detachment, increased interpersonal conflicts and turnover/intent to leave (92, 141). Signs may include frustration and anger at work, irritability, anxiety and panic, overreactions, feeling upset or sad without knowing why, and feeling unable to control one's emotions (86). In human medicine, emotionally exhausted clinicians are less able to engage in positive interpersonal teamwork, which may initiate a vicious cycle of degrading relationships with coworkers (142). The symptomology of burnout overlaps with that of mental health disorders such as depression, anxiety, and suicidal ideation; for example, emotional exhaustion and cynicism are positively related to symptoms of anxiety (72, 143, 144). Recent work also exposed a high degree of overlap between signs of burnout and workplace post-traumatic stress disorder (PTSD) in physicians; bullying and overwhelming work responsibilities were reported most frequently as the inciting traumatic stressors (145, 146). Workers with PTSD experience a deterioration of physical and psychological health, impairment of social and occupational functioning as a result of intrusive memories, avoidance, negative alterations in mood and cognition, and hyperarousal to stressors (145). These other disorders differ from burnout but are frequent comorbidities (72, 143).

In a study of 40 resident physicians, the mean emotional intelligence scores were lower in those with burnout (3.7, SD = 0.33) vs. those without burnout (3.9, SD = 0.16, *p* < 0.02), although measures of clinical competency did not differ between groups (147). Individuals experiencing protracted and excessive workplace stress commonly experience an increase in self-protective reactivity, isolating behaviors, and a deterioration in their own communication skills in a self-perpetuating cycle that impacts teamwork. Clinicians are more likely to experience mental ill-health when they feel isolated or unable to do their job, and when they fear repercussions of help-seeking (148). Individuals with burnout often go unrecognized or “misdiagnosed” by management and peers, and healthcare professionals are very commonly systemically disincentivized to acknowledge that they have problems and to seek assistance. Despite the prevalence of burnout, speaking up or seeking help to deal with work-related stress continues to be perceived, especially within the culture of healthcare, as a sign of weakness or inability to “make it” as a clinician (149, 150). Mental health stigma (151, 152) and self-stigma (153) are greater in veterinary and medical trainees and practicing veterinarians and physicians compared to non-healthcare workers, which may prevent appropriate health care-seeking behavior in these burnout-affected individuals (154, 155). In a recent study of veterinarians, the majority (97%) expressed that treatment helps individuals with mental illness lead normal lives, but a much lower proportion (53%) agreed that people are caring and sympathetic toward individuals with mental illness (155). Negative attitudes toward the presence of social support for mental illness were more likely in veterinarians aged 40–59 years of age (compared to those 20–39 years; 46 vs. 37%, OR = 1.18, *p* < 0.001), in women (compared to men; 75 vs 64%, *p* < 0.001), by solo practitioners (vs. non-solo; 81 vs. 19%, OR = 1.23, *p* = 0.08), in those with evidence of serious psychological distress (vs. without; OR = 1.55, 22 vs. 9%, *p* < 0.001), and by those reporting mental illness after graduating from veterinary school (vs. reporting no mental illness; 42 vs. 58%, OR = 1.66, *p* < 0.001) (155). Physicians are much less likely to seek help for depression or other mental health issues, or to have been taking an antidepressant at the time of suicide compared to non-physician suicides (154). In a study of 516 university hospital physicians reporting recent suicidal thoughts and/or showing other indicators of psychological ill health, a staggering 78% of these distressed physicians had never sought professional help for depression or burnout (156). In this cohort, three work-related characteristics were associated with not seeking needed help for burnout or depression: current involvement in medical research (75%, OR = 3.39, 95% CI = 1.43–8.02), taking night call (75%, OR = 1.86, 95% CI = 0.8–4.32), and being a surgical specialist (38%, OR = 1.18, 95% CI = 0.53–2.63) (156). These types of numbers raise real concern for not only for affected academic clinicians themselves, but also for the implications to patient care, clinical research, and the education of future doctors, both physicians and veterinarians (156).

### 5.4. Resilience

Burnout is not unique to healthcare, providing opportunities to learn from other professions. One of the first investigations on burnout involved air traffic controllers. In this study, mental health problems and hypertension were associated with uninterrupted long shifts, rapidly changing shift patterns, fatigue, inadequate equipment and challenges with human-machine interfaces, poor training, and an unsafe workload (53, 55). Workers who were highly competent and strove hardest to meet internal and external professional ideals under these conditions seemed at greatest risk of burnout, initiating a self-perpetuating cycle of chronic stress, irrespective of their individual resilience (53, 55).

Resilience should be cultivated among all humans for its undeniable value in navigating adversity. However, emphasizing individual resilience in organizational messaging places both the blame as well as the burden of managing workplace stress inequitably on the affected individual, and also distracts from correcting the fundamental sources of pathologic stress (157, 158). Especially for our trainees (students, interns, and residents), this messaging delivers mixed messages. If they experience burnout, they may feel as perceived to be both lacking resilience and failing to take personal responsibility for defining their limits. Conversely, if they do set rational limits for themselves regarding sleep, workload, and stress, they may receive cues that they do not have the required level of professional exceptionalism and willingness to do “whatever is necessary, ” to be a valued practitioner or reach the next steps of their careers.

In a review of resilience in the health professions, the only factors exhibiting strongly supportive association with resilience across all professions studied were female gender and maintaining a work-life balance (159). In other research, the effect of gender difference varies, and different studies of burnout and stress come to differing conclusions, supporting that different hospital environments or work factors (e.g., the impact of specialty type) are more important than individual factors (160). Physicians as a group actually tend to score higher on resilience scales than individuals non-healthcare professions, providing further evidence that clinician burnout occurs *in spite of* the protective effects of resilience (161). Mean resilience scores were higher among 5,455 physicians than the general working population (OR = 6.49 [SD = 1.30] vs. 6.25 [SD = 1.37]; 95% CI = 0.19–0.32; *p* < 0.001) (161). While physicians without overall burnout had higher mean resilience scores than physicians with burnout (OR = 6.82 [SD = 1.15] vs. 6.13 [SD = 1.36]; 95% CI = 0.61–0.76; *p* < 0.001), 29% of physicians exhibiting the highest possible resilience score still exhibited burnout (161).

Viewing burnout as a personal failure or lack of individual resilience is an antiquated misconception that affects its prevention and is therefore harmful to healthcare professionals. It is important to avoid oversimplifying the very complex topic of burnout to personality types, character, or imperfect resilience characteristics that may or may not be exhibited by individuals. Focusing on the person suggests that burnout arises because individuals are unable to adapt to the learning or work environment and does not address the underlying system dysfunctions. “*One of the tragic paradoxes of burnout is that those who are most susceptible seem to be the most dedicated, conscientious, responsible, and motivated. Individuals with these traits are often idealistic and have perfectionist qualities ... Those are the very traits sought by most medical school admission committees, most residency and fellowship training program directors, most patients seeking a physician, and most physicians seeking to hire a new associate*” (162, 163). While understanding impacts of different personality characteristics can provide useful information for individual self-reflection, or to aid a mental health professional in diagnosis and treatment of an individual suffering with mental illness, this does not treat the fundamental cause of burnout. Additionally, if not communicated correctly, organizational emphasis of individual resilience sends the message to affected professionals that they are the problem, that they need to do better at “absorbing negative conditions, ” and that failure to “tough it out” is a sign of weakness; all of which is counterproductive messaging if the goal is to cultivate behaviors that prevent or eliminate professional burnout (164, 165).

### 5.5. Individual recovery

Treatment of the effects of established burnout and its outcomes vary greatly between duration, severity, and a variety of individual factors, and should be directed based on consultation with appropriate healthcare professionals (166). Individual wellness strategies help to manage symptoms of excessive stress and can provide temporary relief, but if the fundamental causes of burnout are not addressed, burnout symptoms often return to pre-intervention levels within a year after interventions are implemented (42, 167). Severe work-related stress is associated with increased risks for long term sick-leave, although affected individuals generally do experience improvements during medical leave (168, 169). Recovery from more severe forms of burnout is possible but requires a frank appraisal of the contributing personal and professional factors, along with deliberate and intentional changes to address them, including reduction in work hours or reassignment of duties (140). Prospective studies in medical students and residents suggest that only ~12–27% of those making appropriate efforts to address the issue will recover fully from more severe forms of burnout within the initial 12 months after identification (140). More severely impacted individuals may require a work hiatus of up to several years and some severely affected individuals never return to work (86).

**Figure 2:**
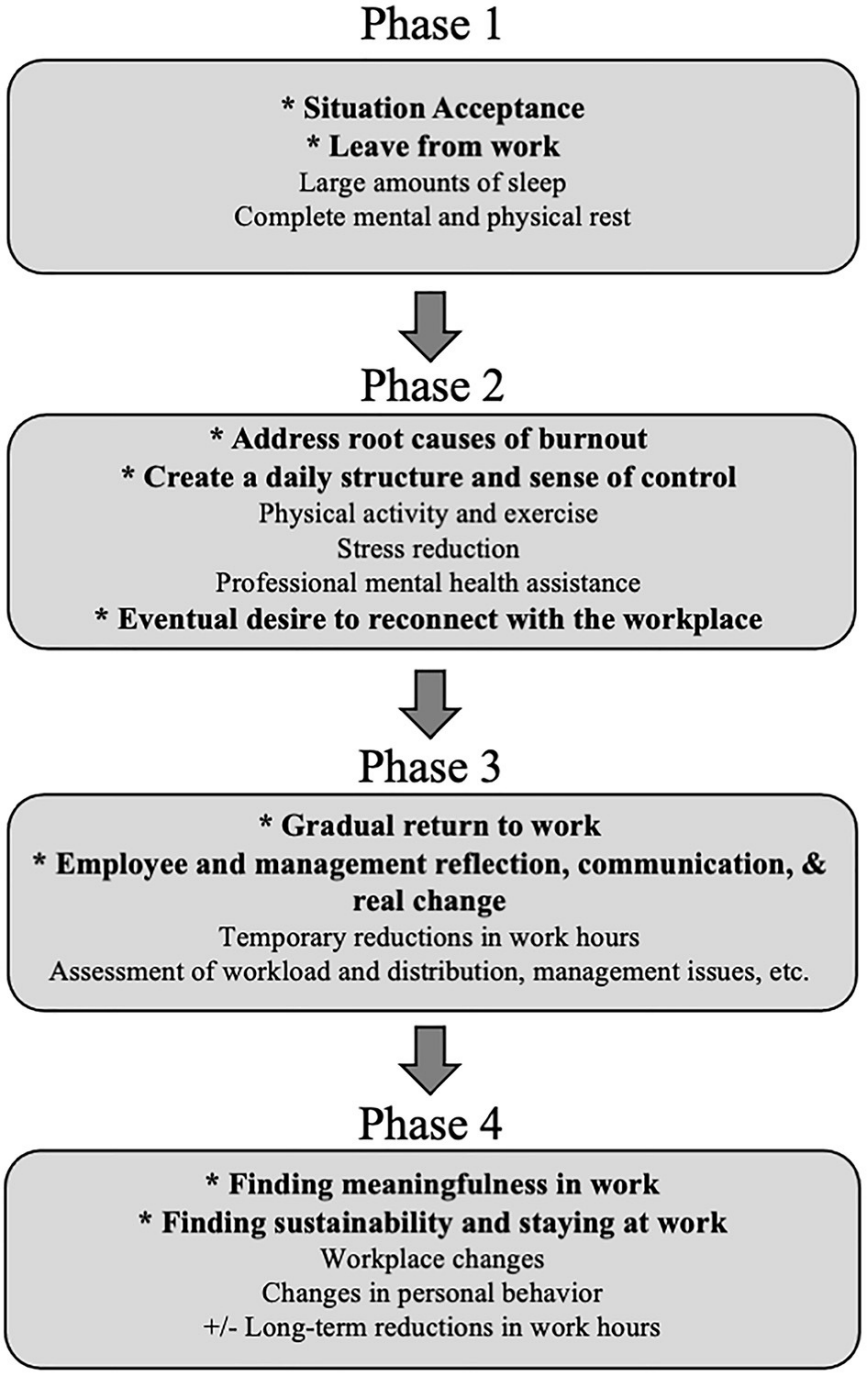
Described phases of recovery from clinical burnout. Adapted from concepts presented by Pijpker et al. (170).

Four stages of recovery from burnout have been described (Figure 2) (170). Studies evaluating successful rehabilitation after burnout emphasize the need to address both personal (e.g., coping skills) and work-related factors (e.g., workload and work environment), while also considering the specific needs of individual employees (42, 171–173). A 25% reduction of weekly work hours for full-time employees has been shown to improve sleep and alertness and reduce stress during both workdays and days off, and these effects were maintained over the 18-month study period (174). This work reduction resulted in decreased total workload and an increase of time spent in recovery activities and supported that ongoing work time reduction may be important for stress reduction and long-term health (175). Individuals can reduce risks and impacts of burnout by finding meaning in work but also re-defining their life priorities and incorporating a philosophy of work-life balance (140). Promoting personal health often requires an uphill battle against a professional culture that puts work first, prioritizes other needs above self, and emphasizes professional over personal achievements (176). Evidence does suggest that the reduction of long-term burnout complaints and the promotion of return-to-work are independent processes, neither of which is well understood (177).

## 6. Occupational stressors in healthcare

Healthcare, including veterinary medicine, is a demanding, high mental load profession that often carries high administrative burdens and job strain. Veterinarians have complex performance requirements for cognition, information processing and decision making, emotion and affect management, and navigation of interpersonal tensions. High-strain work is characterized by high demands and low control; such critical combination generates psychological pressure often leading to fatigue, depression, anxiety, and disease (178). Stress originates from dealing with ambiguity and uncertainty (179). These characteristics are common in healthcare occupations intrinsically fraught with uncertain tasks, tough deadlines, and conflicting requirements. The broad drivers of physician burnout have been described in 7 dimensions by Shanafelt et al. (180) as (1) workload and job demands, (2) efficiency and resources, (3) flexibility/control over work, (4) work–life integration, (5) alignment of individual and organizational culture and values, (6) social support/sense of community at work, and (7) the degree of meaning derived from work (180). The themes are similar to the six mismatches previously identified by Maslach and Leiter et al as the source of burnout in the general modern workplace: (1) work overload, (2) lack of control, (3) insufficient reward, (4) breakdown of community, (5) lack of fairness, and (6) conflicting values (181).

The evolution of modern medical care delivery mechanisms further compounds these factors. Historically, physicians and veterinarians had almost complete control over their independent practices, which likely contributed to reducing job strain to some degree. However, increasing numbers of physicians and veterinarians have shifted from practice ownership to employment in large hospital and corporate organizations. Such setting hinders their authority to resolve issues of chronic stress, excessive job strain and burnout, requiring engagement, approval, and support by organizational leadership (138, 182). Healthcare professionals commonly share a drive to work harder and “get the job done” when faced with adversity, but this apparent asset can lead to pathological altruism (43) while providing a convenient and perhaps often over-exploited path for healthcare organizations to manage resource deficits (183). The resulting dysfunctional professionalism perpetuates maladaptive structures in both veterinary education and practice.

To strive for psychological wellbeing does not imply the requirement for an absence of stress and/or painful emotions at all times. It is when negative or painful emotions become extreme and long-lasting that psychological wellbeing is impacted (184). High levels of occupational stress may be tolerable and even team building in the short term, but they become destructive in the long term. Certain forms of work stress can even result in workplace post-traumatic stress disorder (PTSD), a potentially debilitating condition (146). While the appearance of burnout in an affected clinician might appear sudden, the syndrome does not develop overnight. The emotional exhaustion experienced by individuals or teams likely results from a poor climate which evolves slowly over time and signals chronically insufficient prioritization of employees' health by the organizational management (185). At its root cause, burnout is a systems dysfunction, in which structural or organizational intervention and mitigation of the sources of dysfunctional work environments are imperative for correction (43). The unfortunately high incidence of this problem in the veterinary profession reflects widespread unsustainable structuring of work and long-term inadequate support of the workforce (1, 43, 163).

### 6.1. Unavoidable occupational suffering

It is indisputable that the very nature of veterinary work can cause stress. Stressors inherent to the veterinary profession include life and death decision making, euthanizing animals, economic restrictions of care, adverse outcomes, and client grief (Table 2) (186, 200, 201). The performance of euthanasia by veterinarians is an aspect of practice that differs fundamentally from that of physicians and most other professions, and this is an aspect that crosses species and practice type lines, impacting small animal and large animal practitioners alike. Morality-related stressors such as euthanasia, abusive situations, and economic restrictions of care (among others) can be a source of serious moral distress (Table 1) for veterinarians (200). The magnitude of impact is variable; some studies suggest a high incidence of occupational stress and euthanasia-related strain in animal care personnel (including veterinarians) (202) and in veterinarians dealing with client bereavement (186), but others have found the magnitude and significance of this type of stress to be highly individual (201, 203). Crane et al. found that while such stressors were related to increases in milder expressions of distress, they did not appear to be associated with more severe decrements in psychological wellbeing (201). The same study found that a combination of stressor events and individual tendencies toward perfectionism traits appeared to create vulnerability to moral stressors, and that the moral significance of stressors was associated with an individual's psychological resilience (201). Methods of fostering resilience and coping skills (204) are logical and useful steps in addressing moral stressors and unavoidable suffering. The impacts of moral distress on individuals are not minimized but should also not divert attention from necessary fundamental system reforms.

**Table 2:** Sources of occupational stress pertinent to burnout and the wellbeing of veterinary and other healthcare professionals (2, 4–6, 23, 26, 51, 75, 84, 141, 186–199).

**Avoidable/modifiable occupational stressors**			
**Organizational infrastructure and logistics**	· Physical hazards	**Schedule**	· Fast paced workdays
	· Workplace injuries		· Long work hours
	· Workplace ergonomic issues		· Work-related sleep deprivation
	· Insufficient, substandard or lack of work tools and equipment or resources		· Night schedule or on-call
	· Type of practice/specialty		· Weekend schedule or on-call
**Work assignment**	· Excessive workload or high-level responsibilities		· Lack of control over one's daily schedule
	· Frequently shifting/changing work responsibilities		· Insufficient mental breaks, hydration, nutrition
	· Work compression	**Management**	· Unclear work role expectations
	· Administrative task load		· Contradictory instructions/expectations from supervisors
	· Technology, poor electronic medical record systems		· Promotion of individual rather than collective achievements
	· Lack of autonomy or authority		· Competitive work environment; poor teamwork
	· Poor alignment of responsibility and authority		· Real or perceived inequity in promotion or compensation
	· Role conflicts between clinical service, research, education and administration		· Bias or discrimination (gender or otherwise)
**Colleagues/Co-workers**	· Poor functionality of team structures		· Harassment (sexual or otherwise)
	· Conflicts with peers		· Poor leadership
	· Competing interests among the team		· Poor teamwork
	· Workplace bullying or mobbing		· Insufficient positive feedback
	· Absence of social support at work		· Moral injury or distress
**Client/patient issues**	· Client demands/expectations/complaints, high volume of requests for non-billable communications		· Poor leadership cohesion and shared vision, power dynamics, hierarchy problems
	· Caregiver burden, emotional labor of clinical empathy		· Poor organizational communication
	· Client harassment or violence		· Excessive work pressure
	· Social media and cyberbullying	**Culture issues**	· Presenteeism
	· Medical error or near miss		· Workism
	· Fear of malpractice litigation		· Hidden curriculum
	· Compassion fatigue		· Indefatiguable clinician construct
**Unavoidable occupational stressors**			
**Organizational**	· Competition from other veterinary practices	**Client/patient issues**	· Client demands/expectations/complaints
	· Accreditation, licensure, and certification requirements		· Ethical dilemmas regarding treatment options
	· Insurance requirements		· Lack of client compliance
	· National and state policies and laws		· Suspicion of patient/animal abuse
	· Resource limitations		· Client economic limitations
	· Practice management responsibilities for owners		· Experiencing adverse treatment outcomes
· Client grief		
· Euthanasia		

The existing literature suggests that the most important stressors that veterinarians experience are related to workload, emotional demands, issues with clients, issues with coworkers, financial worries, negative work-home interactions, and high responsibilities, some of which are still intrinsic and unavoidable (12). The most predictive work-related factors for burnout and suicide ideation in veterinarians have been identified as workload (*p* < 0.01), client issues (*p* < 0.05), financial issues (*p* < 0.05), and strong feelings of responsibility (*p* < 0.01) (12). In a study of veterinarian wellbeing relative to client grief, a strong association was found between the total compassion fatigue score and hours worked, suggesting that the magnitude of impact of moral stress experienced may be amplified by workplace factors (186). Employers and educational organizations bear responsibility for worsening unavoidable suffering if they accept or even actively create institutional cultures where occupational stress is worsened by negative or outdated policies, work structure, and behavioral patterns (187). Personal stressors can also add to a veterinarian's overall stress load and in doing so, contribute to the development of burnout (Table 3).

**Table 3:** Sources of personal stress pertinent to veterinary and other healthcare professional burnout and wellbeing (4–6, 23, 75, 84, 141, 187, 188, 190–195, 197).

**Personal stressors**
· Career stage, especially recent qualification or entry into the profession
· Poor work-life integration
· Personality traits of overachievement, perfectionism, neuroticisim, idealistic self-expectation, and need for recognition
· Imposter syndrome, perceptions of inadequacy or reduced competency in one's job
· Trait of people pleasing behaviors (including suppression of one's own needs)
· Feeling irreplaceable in an area or areas of work
· Choice of work as one's only meaningful activity
· Choice of work as a substitute for social life
· Loss of meaning or joy in work
· Preference to work with animals/social isolation/poor social support
· Lack of personal skills in communication, coping, delegation, organization, or teamwork
· Alignment of societal expectations and gender/race/sexual orientation
· Educational debt or other personal financial issues
· Personal loss/death/divorce
· Personal caregiver responsibilities and other family situations
· Personal life-related sleep loss
· Stigmatization of mental illness
· Insufficient exercise
· Injury/chronic pain/chronic illness

### 6.2. Avoidable occupational suffering

#### 6.2.1. Workload, job demands, and work complexity

Client complaints and high workload are ranked among the greatest occupational stressors for veterinarians (188, 189, 205, 206). Workload can be conceptualized in two different ways: either as (1) the work or task load that is performed or capable of being performed within a specified time, or (2) as the mental, physical, or physiologic effects experienced by the worker as a result of the work (as defined by validated worker self-report tools, physiological measures such as heart rate and heart rate variability, or quantitative metrics related to the tasks being performed) (207–209). In a national study of over 5000 physicians, it was observed that every 10% reduction in task load was associated with 33% lower odds of experiencing burnout (OR = 0.67, 95% CI = 0.65–0.70, *p* < 0.0001), and on multivariate analysis, task load was a significant predictor of burnout independent of age, gender, specialty, hours worked, and practice setting (79). Cognitively taxing environments that require constantly complex and consequential decision-making result in a state of cognitive exhaustion that further impacts individual cognition and task completion comparable to the equivalent tolls of losing an entire night of sleep, being an alcoholic, or losing ~13 IQ points (210, 211).

In addition to issues of job demands during work hours, work availability after hours is an important factor in the development of burnout (212). Psychological detachment or the ability to mentally disengage (refrain from job-related activities and thoughts during non-work time) is both a mediator and a moderator in the relationship between job stressors, job strain and poor wellbeing (213). Overall, research demonstrates that in general occupational stressors such as workload predict low levels of psychological detachment (213). A lack of detachment in turn predicts high occupational strain levels and poor individual wellbeing (e.g., greater burnout and lower life satisfaction) (213). This issue is likely to be particularly relevant in veterinary practice situations requiring after-hours patient management that is dependent upon clinicians maintaining on-call status for return to work, telephone consult availability, or for veterinarians who may be awoken during the night for patient updates, but other work factors including at-home record management or email communication also inhibit the ability of veterinarians to disengage and detach from work sufficiently to achieve fundamentally necessary physiologic recovery.

An underappreciated aspect of modern healthcare workloads is that of information chaos, comprised of various combinations of information overload, information underload, information scatter, information conflict, and erroneous information (214, 215). In addition to information chaos, effects of communication overload impact medical workloads (216). In a cohort of 101 emergency physicians, there was overall agreement that information overload negatively impacts work, and the problem has been increasing over time (217). The three main reasons identified in that study were: the “always available culture”, email handling, and multidisciplinary communications (217). In physicians, the electronic health record (EHR) is commonly a major source of dissatisfaction and burnout (218, 219); veterinary specific data is not available, but anecdotally, veterinary practice is also increasingly computerized. Commonly identified associations between EHR-related burnout in healthcare providers include message and alert load, time spent on EHRs, organizational support, EHR functionality and usability, and general use of EHRs (220). Well-functioning electronic health records can foster better communication between clinicians and patients (or veterinary clients) and facilitate better access to patient data and quality care, but over-burdensome documentation requirements may make otherwise well-designed EHRs frustrating to use simply because of overbearing amounts of time needed to complete the record requirements (221, 222). Compared with those who reported having sufficient time for documentation at work, physicians reporting poor/marginal time exhibited nearly three times the odds of burnout (OR = 2.8, 95% CI = 2.0–4.1, *p* < 0.0001) (223). It has been reported that for every hour spent with a patient, physicians commonly spend an additional 1–2 hours on the EHR at work, with additional time spent on the EHR at home after work hours (224, 225). Physicians who reported moderately high to excessive time spent on EHRs work at home were at greater risk of burnout (OR = 1.9, 95% CI = 1.4–2.8, *p* < 0.0001) compared to those who performed minimal/no EHR use at home (223). The transformation from paper-based medical records to EHRs also resulted in an expanded scope for these documents to serve administrative and legal purposes, and in some environments, those of research and education (226). This has resulted in lengthy and complex records with additional non-clinical text, and the problem is further exacerbated by issues of “note bloat,” where templates and copy-paste actions may worsen the problem of information overload and reduce efficiency of communication (226). Extra effort is frequently required to search through “note bloated” charts for specific information, or to clarify conflicting documentation, which increases cognitive load. While technology does bring many practice and patient benefits, these and other forms of technology including excessive email (227, 228) and the use of increasingly complicated devices in medical care (208, 229, 230) do lead to work intensification and task overload, and in doing so contribute to burnout.

#### 6.2.2. Human factors, ergonomics, and occupational health

Veterinarians are at risk for a variety of occupational health issues including zoonotic infectious disease, chemical, and ionizing radiation exposures, physical injuries (e.g., needle stick injury, bites, cuts, and others), skin irritation, and a variety of types of musculoskeletal discomfort associated with poor ergonomics and strenuous work postures (231–240). Tasks considered by veterinarians most likely to lead to musculoskeletal discomfort were lifting, surgery, rectal palpations, and animal handling (241). In a cohort of German veterinarians, the majority of accidental injury in the distal upper extremities were caused by animals (19%) compared to other factors (9%) (242). In a variety of different veterinary studies, musculoskeletal discomfort in the 12 months prior was reported in the lower back (63–73%), upper back (34%), neck (57–67%), shoulder (52–61%) elbow (24.5%), or hand (34.5%), or legs/feet (>60%) (242–245). The reported prevalence of musculoskeletal discomfort in bovine practitioners was particularly high (90% 12-month, and 97% lifetime rates, respectively) (246).

Performing surgery is a well-documented occupational risk for a variety of types of musculoskeletal discomfort and injury, and operating room ergonomics and workflow are often poorly considered (247, 248). Veterinarians performing soft tissue and orthopedic surgery commonly experience demanding, static postures. Ninety percent of veterinary surgeons performing laparoscopic surgery reported that the surgical procedures caused or exacerbated pain that they experienced in the neck (81%), back (77%) and shoulders (75%), either during or after the procedure, including pain experienced at home (54%) (249). In another recent study, similar to physician surgeons, 93% of specialist veterinary surgeons reported experiencing musculoskeletal discomfort associated with surgery, and discomfort was reported regardless of practice emphasis or procedure type (250, 251). Over 85% of respondents showed more than some concern regarding career longevity due to musculoskeletal pain (250). Among veterinarians performing spays and neuters, 77% reported hand or wrist pain (right thumb and/or thumb base (50%) and the right wrist (38%) and 93% reported body pain (lower back (77%), shoulders (73%), and neck (72%) (247).

A recent survey of veterinarians and veterinary technicians in the U.S., >85% of participants reported that work activities exacerbated discomfort in at least one musculoskeletal region (244). However less than one-third of the participants reported symptom-related lost work time (244). These combinations of a high prevalence of musculoskeletal discomfort and low amount of lost work suggests that many individuals are working in pain (244). Among physician surgeons, experiencing physical pain at work is well-documented to correlate positively with burnout and to be inversely associated with professional satisfaction and happiness (252–255). Conversely, while most veterinarians perceive the causes of work-related musculoskeletal discomfort to be related to physical rather than psychosocial factors (241), correlations between musculoskeletal discomfort and psychosocial risk factors have been identified (243, 256), supporting that workplace issues of the types that lead to burnout contribute more to the development of musculoskeletal discomfort among veterinarians than previously recognized. Importantly, chronic occupational musculoskeletal pain can be both a cause and an effect of work-related stress. Factors increasing the odds of musculoskeletal discomfort requiring time off work for clinical veterinarians were: 10 year increases in age (OR 1.26, 95% CI 1.05–1.52), work involving awkward grip and hand movements (OR 12.91, 95% CI 3.46–4.21) and those who were dissatisfied with the level and difficulty of their work (OR 2.27, 95% CI 1.11–6.56) (245). These are serious occupational health issues that contribute to burnout, and the high prevalence rates reported support the need for veterinary profession-specific research on direct ergonomic assessments and work practice interventions including improvements in equipment, processes, and procedures in veterinary clinical practice.

#### 6.2.3. Maladaptive professionalism

A variety of maladaptive constructs are deeply embedded in healthcare culture that influence decisions of organizational logistics and that are reinforced and perpetuated by the hidden curriculum, such as presenteeism, pathological altruism, the conflation of sleep loss to work ethic, the construct of the indefatigable clinician, and the equation of excessive duty hours with professional dedication (49, 50). Presenteeism and neglect of personal health are examples of maladaptive behaviors that not only reduce the wellbeing of healthcare workers, but also the quality and safety of patient care (257, 258). Ill employees exhibit reduced capability and productivity, and the act of working while ill further drains mental energy reserves that cannot be quickly recovered the following day (259). Workplace factors that contribute to illness-related presenteeism include specialized roles, physically demanding work, distribution of work tasks within the workplace, anticipated staff shortage exacerbations on coworker stress, concerns for job security, fear of disciplinary action, job identity in relation to self-image, social dynamic of the workplace and fear of judgment by colleagues, a feeling of professional and/or moral obligation to continue working despite illness, and belonging to a profession with long hours and/or high rates of stress and burnout (51, 260). Presenteeism has clearly been associated in a variety of studies with occupational stress and exhaustion as well as anxiety and depression (261–263). Exhaustion and presenteeism are reciprocal; when employees experience exhaustion, they mobilize compensation strategies to be present, which ultimately increases their exhaustion, a cycle that leads to burnout (264).

A long-standing culture of undervaluing clinician rest is an important contributor to the development of burnout, and also hampers recovery of affected individuals once established (49, 50, 97, 99, 265). Sleep deprivation and burnout are distinct, yet intimately associated manifestations of a healthcare system designed for the wellbeing of patients and/or administrative or client convenience, but without consideration for the wellbeing of the healthcare provider. Jobs that regularly bleed into nights and weekends and/or cause circadian disruption via on-call duty and extended or poorly scheduled work shifts are associated with extremely high work stress (49, 266, 267). Occupational sleep deficiencies may potentially be associated with long-term individual impacts, as physicians with chronic on-call duties of ~5–6 24-hour shifts/month exhibited baseline evidence of genetic damage (*d* = 1.48, *p* = 0.0001) including DNA breaks (*d* = 0.87, *p* = 0.0018), as well as impaired DNA repair (*d* = 1.47, *p* = 0.0001) after a night of acute sleep deprivation when compared to participants who did not work extended hours (268). On-call shifts have a particularly negative impact on veterinarian job satisfaction, wellbeing, and personal relationships, especially in female associates (269). Working ≥40 h/week, and perceived staffing shortages are also associated with high risks of burnout (190). Extended work schedules are often suggested to be necessary to ensure continuity of care and thus lower risks of medical errors (270). Indeed, poorly managed care transitions increase the risks for medical errors; however, improvements in handover techniques and process mitigate these effects (50). This argument also ignores the well-known adverse effects on clinician performance and the degradation of clinician wellbeing that is associated with extended duty hour shifts or repeated work shifts without days off; effects that occur even in the absence of sleep deprivation or circadian cycle disturbances (50). When schedules are actively managed using structured handovers, there do not appear to be differences in quality of care between extended schedules and those with reasonable work breaks (50). In a study of 45 physicians caring for 1900 patients in 5 ICUs, patient mortality was non-significantly higher (OR = 1.43, *p* < 0.12) for clinicians on a continuous schedule (14 consecutive day shifts) compared to those on an interrupted schedule (weekday coverage with weekend cross-coverage by colleagues), but those working under the continuous schedule experienced significantly greater burnout, work–home life imbalance, and job distress (271).

#### 6.2.4. Work climate and team environment

Overall organizational climate can be reflected in healthcare professional satisfaction with the work environment and reduced burnout (272, 273). A longitudinal survey in a sample of practicing primary care physicians found that organizational values dissonance along with workload were among the largest drivers of burnout (274). Perceptions of low organizational support, organizational politics or insufficient resources may increase risks of burnout (275–278). Psychosocial aspects of the work environment, such as a lack of perceived fairness in the organization, conflicting demands, and unreasonable expectations, feeling unappreciated, low social support, workplace bullying/mobbing, work–family conflict, and low job control, are associated with poor health as strongly as other more easily measured workplace risks such as shift work, long work hours, and over-time (61, 191). A perceived lack of support at work is also associated with high risk for burnout (190). The effectiveness of a veterinary team can profoundly influence the individual levels of job satisfaction and burnout (60). Coordinated veterinary team environments are associated with increased professional efficacy and decreased cynicism (*p* < 0.05), whereas toxic team environments are negatively associated with job satisfaction (*p* < 0.03) and positively associated with exhaustion (*p* < 0.05) and cynicism (*p* < 0.02) (60).

Workplace bullying is described in every profession, and healthcare, including veterinary medicine, is not immune. This negative behavior is referred to by a variety of labels including mobbing, incivility, harassment, emotional abuse, ostracism, and abusive supervision. It may vary in intent, can arise from personality or work-related factors and stressors (such as poor management, job insecurity, workload, role conflict/ambiguity, and cognitive demands of the job), and a poor work environment often fosters bullying (279, 280). Workplace bullying may result from internal competition, which can become exacerbated in situations of resource limitations (281). As a form of ongoing negative behavior which requires significant energy and redirected effort investment from job duties by an affected individual, bullying becomes a serious job demand for affected individuals and leads to burnout (282).

The effects of both client-originating and colleague-originating incivility in veterinary practice have been reported, with incivility correlating positively with mental health status and burnout (*p* < 0.001), and negatively with job satisfaction (*p* < 0.001) (283, 284). A recent meta-analysis reported a mean pooled prevalence of bullying in the human healthcare workplace of 26% (range, 4–87%) (285) and another systematic review reported a prevalence of bullying among surgeons between 18 and 49% (286). Experiencing bullying is a major work stressor, with destructive consequences for recipient employees, causing frustration, helplessness, negative emotions such as sadness, anger, resentment, and fear, difficulty concentrating, lower self-esteem and self-efficacy, reduced job satisfaction, physical health problems, severe depression, anxiety, PTSD symptoms, suicide ideation, and burnout (282, 285, 287–291). Injustice and unfairness are both serious risk factors for burnout (73). Bullying breaks psychological contracts, creating an unjust workplace, and becomes a negative environmental contagion in which victims of workplace bullying are themselves more likely to exhibit problem behaviors as a consequence of being bullied (279, 288, 292, 293). Of the negative emotions generated by bullying, sadness has been shown to impact individual health, while anger and fear lead to moral disengagement and subsequent workplace misbehavior by the victim themselves (292).

#### 6.2.5. Financial compensation

In general, financial stress of any kind contributes to burnout, especially high levels of educational debt and pay disparities or inequities (129, 294). Greater educational debt was associated with personal life dissatisfaction (*p* = 0.032) and career regret (*p* < 0.002), among physicians, and 76% of this physician cohort agreed that financial stress was related to their feelings of burnout (295). Among other considerations of work-life balance and financial constraints, educational and personal debt have emerged as important factors in physician choices of career type (academia vs. private practice) and specialization (296). Concerns about future earnings was an independent predictor of burnout among another cohort of 700 physicians (275). As a threat to both financial and mental health, educational debt load is a serious issue for healthcare professionals, especially veterinarians (1, 6). While veterinarians and physicians on average both carry serious educational debt, the average debt to income ratio for veterinarians is approximately twice that of physicians (debt:income = 90% for physicians, debt:income = 188% for veterinarians), and over time, the increases in debt have far outpaced increases in income for veterinarians (297). Total annual income was inversely related to burnout scores in a recent study (6). The issue of the debt-to-income ratio was also highlighted in a 2019 veterinary survey where 40% (1,161/2, 874) of respondents reported either high debt or low pay as a reason that they would not recommend the veterinary profession to others (1).

Compensation structures that value quantity over quality increase the risk of burnout within a practice. Productivity-based compensation often leads to overwork and/or shortening the time spent per patient, which in turn leads to increased burnout (298). Depending upon the system and compensation plan specifics, these types of incentivization structures can also contribute to an internally competitive environment related to salary, equipment needs, schedule and caseload. This environment not only leads to stress on interpersonal work relationships but also can influence patient care decisions. These compensation structures too often only reflect the volume of patients seen, and other fundamentally necessary activities that do not directly generate revenue are functionally uncompensated, including client and peer-to-peer phone calls, filling prescriptions, reviewing test results, medical record review and charting, on-call time, mandatory meetings, and continuing education (299). In addition to increased burnout risks, moral injury may be experienced by healthcare providers when there is a direct conflict between their desire to meet patient needs and provide optimal care, vs. the direct and indirect effects of business-driven healthcare environments. Incentive based compensation based entirely on an individual's billings was associated with 37% increased odds of burnout in a study of 7,900 physicians (300).

## 7. Discussion

Awareness of the pathogenesis and pathophysiology of burnout and the effects on the individual described here highlights wide-ranging implications for the profession's methods of practice and the wellbeing of the current and future generations of veterinarians. While a fundamental limitation of this review is the relative dearth of veterinary-specific data on the topics presented here that would permit true profession-specific systematic or meta-analysis at the level of topical depth that we have presented here, a great deal of important and relevant information can be drawn from the experiences of other healthcare providers, and these data are valuable. This review does also deliberately focus more heavily on what we categorize as avoidable occupational suffering, and less on the causes of occupational stress that are often unavoidable or intrinsic to veterinary care. While intrinsic or unavoidable occupational stressors can secondarily contribute to burnout, they more directly result in moral injury and secondary traumatic stress, issues that are no less important, but that require different solutions than those of most causes of burnout. While unavoidable occupational suffering is important and its effects on veterinarian wellbeing is not minimized, the primary goal of these companion manuscripts is to identify and discuss fundamentally avoidable causes of occupational suffering in the profession (or those that would benefit from improved workplace management and professional culture changes) as a lead-in to discussions of systemic and occupational solutions for burnout and improvements in veterinarian wellbeing. The professional and personal demographics of burnout in veterinary and human healthcare, the impacts of burnt-out individuals on the workplace, general burnout mitigation concepts, and ideas for workplace strategies relevant to different veterinary systems will be discussed in a companion review (45).

## Author contributions

MS conceived and designed the review and wrote the first draft of the manuscript. DG provided line editing. All authors contributed to analysis and interpretation, contributed to critical revisions for intellectual content, read, and approved the submitted version.
